# Part II: consensus statements and expert recommendations for BRCA-associated breast cancer in the Asia-Pacific region: clinical management

**DOI:** 10.3389/fonc.2025.1507840

**Published:** 2025-06-23

**Authors:** Yeon Hee Park, Soo Chin Lee, Christian F. Singer, Judith Balmaña, Rebecca Alexandra Dent, Veronique Kiak-Mien Tan, Nadia Ayu Mulansari, Mastura Md. Yusof, Frances Victoria F. Que, Yen-Shen Lu, Napa Parinyanitikul, Cam Phuong Pham, Nur Aishah Taib, Sun-Young Kong, Yoland Antill, Hee Jeong Kim

**Affiliations:** 1 Division of Haematology-Oncology, Department of Medicine, Samsung Medical Centre, Sungkyunkwan University School of Medicine, Seoul, Republic of Korea; 2 Department of Haematology-Oncology, National University Cancer Institute, Singapore, Singapore; 3 Department of Obstetrics and Gynaecology, Comprehensive Cancer Centre, Medical University of Vienna, Vienna, Austria; 4 Department of Medical Oncology, University Hospital Campus Vall Hebron, Barcelona, Spain; 5 Division of Medical Oncology, National Cancer Centre Singapore, Singapore, Singapore; 6 Department of Breast Surgery, Division of Surgery and Surgical Oncology, National Cancer Centre Singapore, Singapore, Singapore; 7 Haematology-Medical Oncology Division, Internal Medicine Department, Cipto Mangunkusumo National General Hospital/Universitas Indonesia, Jakarta, Indonesia; 8 Picaso Cancer Centre, Hospital Picaso, Petaling Jaya, Selangor, Malaysia; 9 Department of Internal Medicine and Oncology, St Luke’s Medical Center, Quezon City and Global City, Metro Manila, Philippines; 10 Department of Oncology, National Taiwan University Hospital, Taipei, Taiwan; 11 Medical Oncology Unit, King Chulalongkorn Memorial Hospital, Bangkok, Thailand; 12 The Nuclear Medicine and Oncology Center, Bachmai Hospital, Hanoi, Vietnam; 13 Nuclear Medicine Department, Hanoi Medical University, Hanoi, Vietnam; 14 Oncology and Nuclear Medicine Department, University of Medicine and Pharmacy, Vietnam National University, Hanoi, Vietnam; 15 Department of Surgery, Faculty of Medicine, University Malaya, UM Cancer Research Institute, Kuala Lumpur, Malaysia; 16 Department of Laboratory Medicine and Genetic Counselling Clinic, National Cancer Center, Goyang, Gyeonggi-do, Republic of Korea; 17 Familial Cancer Centre, Royal Melbourne Hospital, Melbourne, VIC, Australia; 18 Faculty of Medicine and Health Sciences, Monash University, Melbourne, VIC, Australia; 19 Division of Breast Surgery, Department of Surgery, Asan Medical Center, University of Ulsan College of Medicine, Seoul, Republic of Korea

**Keywords:** BRCA germline pathogenic variants, early breast cancer, HER2, PARP inhibitors, triple-negative breast cancer

## Abstract

**Introduction:**

Existing guidelines have practical gaps in decision and treatment sequencing for BRCA germline pathogenic variant breast cancers. This paper aims to develop clinical-practice consensus guidelines to address these gaps in the clinical management of BRCA germline pathogenic variants-associated breast cancer in the Asia-Pacific region.

**Methods:**

An expert panel of 16 medical oncologists, geneticists, and breast cancer surgeons from the Asia-Pacific region arrived at 25 statements. The high level of consensus of statements was considered at ≥75%. A survey of 134 healthcare practitioners, breast cancer surgeons, geneticists, oncologists, molecular biologists/pathologists explored the real- world practices in the Asia-Pacific region.

**Results:**

A consensus was reached for 80% of the statements (20/25) and aligned with the international guidelines. A significant gap was observed between real-world practices and the recommendations of the steering committee members in discussing contralateral risk reducing mastectomy with the patients as a part of standard practice, considering poly ADP-ribose polymerase inhibitor (PARPi) + immunotherapy for early triple negative breast cancer (eTNBC) patients with BRCA variants who don’t achieve pathological complete response after neoadjuvant chemotherapy + immunotherapy, use of adjuvant PARPi in patients with BRCA germline pathogenic variants in eTNBC who have achieved pathological complete response from neoadjuvant therapy, and preference for endocrine therapy + PARPi over endocrine therapy + cyclin-dependent kinase 4/6 inhibitors (CDK4/6i) as escalated adjuvant treatment for BRCA pathogenic variants with high-risk hormone receptor positive/human epidermal growth factor receptor 2 negative (HR+/HER2-negative) early breast cancer.

**Conclusion:**

Testing for BRCA germline pathogenic variants should be expanded to include all young patients with breast cancer. Patients with BRCA germline pathogenic variants should undergo genetic testing before surgery as it can impact surgical intervention decisions and further systemic treatment. The use of neoadjuvant platinum agents in chemotherapy increases the pathological complete response rate. Adjuvant PARPi is preferred over CDK4/6i as escalated treatment in patients who are HR+/HER2-negative.

## 1 Introduction

Breast cancer (BC) has long been and remains a major public health problem (1). Hereditary BC constitutes 5%–10% of all BC cases and 15%–40% of these cases are caused by pathogenic variants of the BC genes 1 and 2 (*BRCA1* and *BRCA2)* (2, 3). The *BRCA1* germline pathogenic variant is more likely to be associated with triple-negative BC (TNBC) and the *BRCA2* germline pathogenic variant is more often associated with hormone receptor (HR)-positive BC (4). The reported prevalence of *BRCA1/2* germline pathogenic variants in Asian patients with familial BC ranges from 8·0% to 31·8% and in those with young-onset BC, it ranges from 2·8% to 21·4% (5).

The prevalence of *BRCA1/2* germline pathogenic variants varies among ethnic groups and geographical areas; however, most *BRCA1/2* germline pathogenic variant patients are young (<45 years of age), have aggressive disease characteristics, and have a family history of *BRCA*-related tumours (3, 6). Additionally, patients with *BRCA1/2* germline pathogenic variants have a risk of developing contralateral breast cancer (CBC) (3). For the individual, identifying these pathogenic variants can impact screening strategies, risk reduction measures, surgical options, radiation, and systemic therapies to improve BC prognosis (7).

Treating hereditary BC is more challenging than treating sporadic BC. Optimal surgical management remains an individualised and hotly debated topic, as *BRCA1/2* germline pathogenic variant carriers often need aggressive surgical interventions for not just therapeutic but also risk reduction purposes (2). Over the past century, the treatment of BC has changed dramatically from a surgical-only approach to a multidisciplinary one that can include radiotherapy, chemotherapy (CT), endocrine therapy (ET), targeted therapy, and immunotherapy (IO) (1). Despite these advances, treatment options for patients with *BRCA* germline pathogenic variants BC are still limited. ET is crucial in managing HR-positive BC-carrying *BRCA* germline pathogenic variants, while CT has been the cornerstone of treatment for patients with *BRCA* germline pathogenic variants TNBC (4). Even in contemporary practice with neoadjuvant CT in conjunction with an immune checkpoint inhibitor (ICI), the likelihood of recurrence for patients with TNBC who do not achieve pathological complete response (pCR) remains high. As of now, patients with TNBC still face the worst prognosis among all clinical subtypes (8). More recently, effective biomarker-targeted therapies, such as poly (ADP-ribose) polymerase inhibitors (PARPis), have been added to the physicians’ armamentarium for treating BC in the *BRCA1/2* germline pathogenic variant (2, 9).

On one hand, there are unmet needs for new treatments for high-risk patients, while on the other, there is a significant risk of overtreating patients at a lower risk of relapse (10). In this complex and rapidly changing environment, not all clinical scenarios can be explicitly informed by data from randomised trials. There are multiple challenges and heterogeneities in BC management in the Asia-Pacific region, such as a lack of effective screening programmes, delays in seeking healthcare by patients, high attrition rates in patients seeking healthcare due to unacceptable out-of-pocket expenditure, and a shortage or skewed distribution of limited resources. The frequent introduction of new drugs for BC treatment into the healthcare system leads to complexity in deciding an optimal therapeutic course. Hence, careful considerations are mandated before drugs are chosen for therapy (11).

There are multiple gaps in the diagnosis and treatment of BC in real-world scenarios that healthcare practitioners (HCPs) often face in their routine practice. The available evidence is often not sufficient to manage these practical gaps. Regional differences in the epidemiology of BC, available resources, and the scarcity of empirical data in the Asia-Pacific region indicate the need for consensus guidelines and expert opinions. This consensus paper aims to provide practical, real-world recommendations for the treatment and management of high-risk human epidermal growth factor receptor 2 (*HER2*)-negative early BC (eBC) carrying *BRCA* germline pathogenic variant in the Asia-Pacific region. The paper is a continuation of a consensus paper that defined the eligibility criteria for genetic counselling and the optimal time to test for *BRCA* germline pathogenic variants in *HER2-*negative eBC; the paper also elucidated clinical risk stratification guidelines for surgical and therapeutic decisions.

### 1.1 Objectives

The objective of this consensus paper is to establish clinical recommendations for decisions in (a) surgical interventions in *HER2*-negative eBC carrying *BRCA* germline pathogenic variants, (b) treatment sequencing in HR-positive *HER2*-negative eBC or early TNBC (eTNBC) that are *BRCA* germline pathogenic variants, and (c) management of metastatic *HER2*-negative BC carrying *BRCA* germline pathogenic variants.

## 2 Methodology

**Figure 1:**
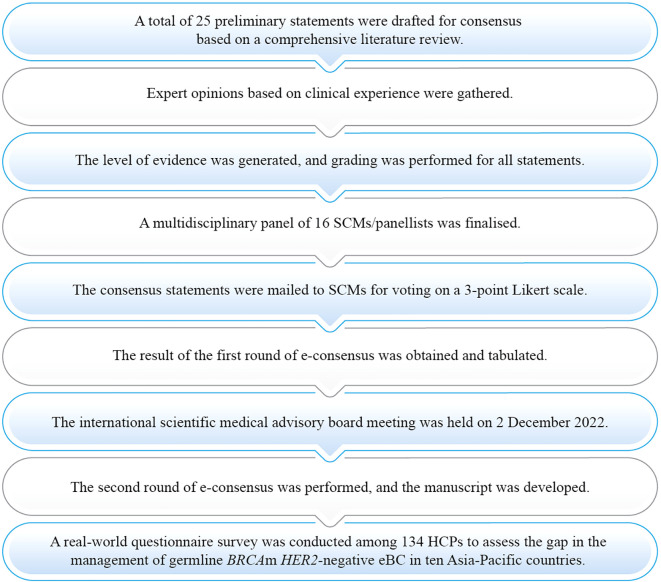
Process of consensus development eBC, Early breast cancer; HCP, Healthcare practitioner; *HER2,* Human epidermal growth factor receptor 2; m, Mutation; SCM, Steering committee member.

A modified Delphi technique was conducted with two online surveys and one scientific advisory board meeting. A set of 25 preliminary statements was drafted by 16 steering committee members (SCMs) and their responses were obtained after three rounds of modified Delphi (**Figure 1**).

The details of the SCMs involved in this paper are provided in **Supplementary Table S1**. For each round of modified Delphi techniques, the participant count remained constant, with 16 SCMs involved. However, to determine the level of consensus, individuals who abstained due to a perceived lack of expertise were not counted in the denominator for all statements. Consequently, statements with denominators less than 16 do not include responses from experts who cited a lack of expertise as their reason for abstaining. The details of the search strategy used for the literature search to develop consensus statements are presented in **Supplementary Table S2**. The details of the response for each statement are presented in **Supplementary Table S3**. A real-world survey was conducted among HCPs to understand gaps in the clinical practices for managing BC in the Asia-Pacific region. The detailed methodology has been presented in the previous manuscript. (Consensus Statements and Expert Recommendations for *HER2-*Negative Early Breast Cancer in the Asia-Pacific Region: Diagnosis and Risk Assessment).

## 3 Results

**Figure 2:**
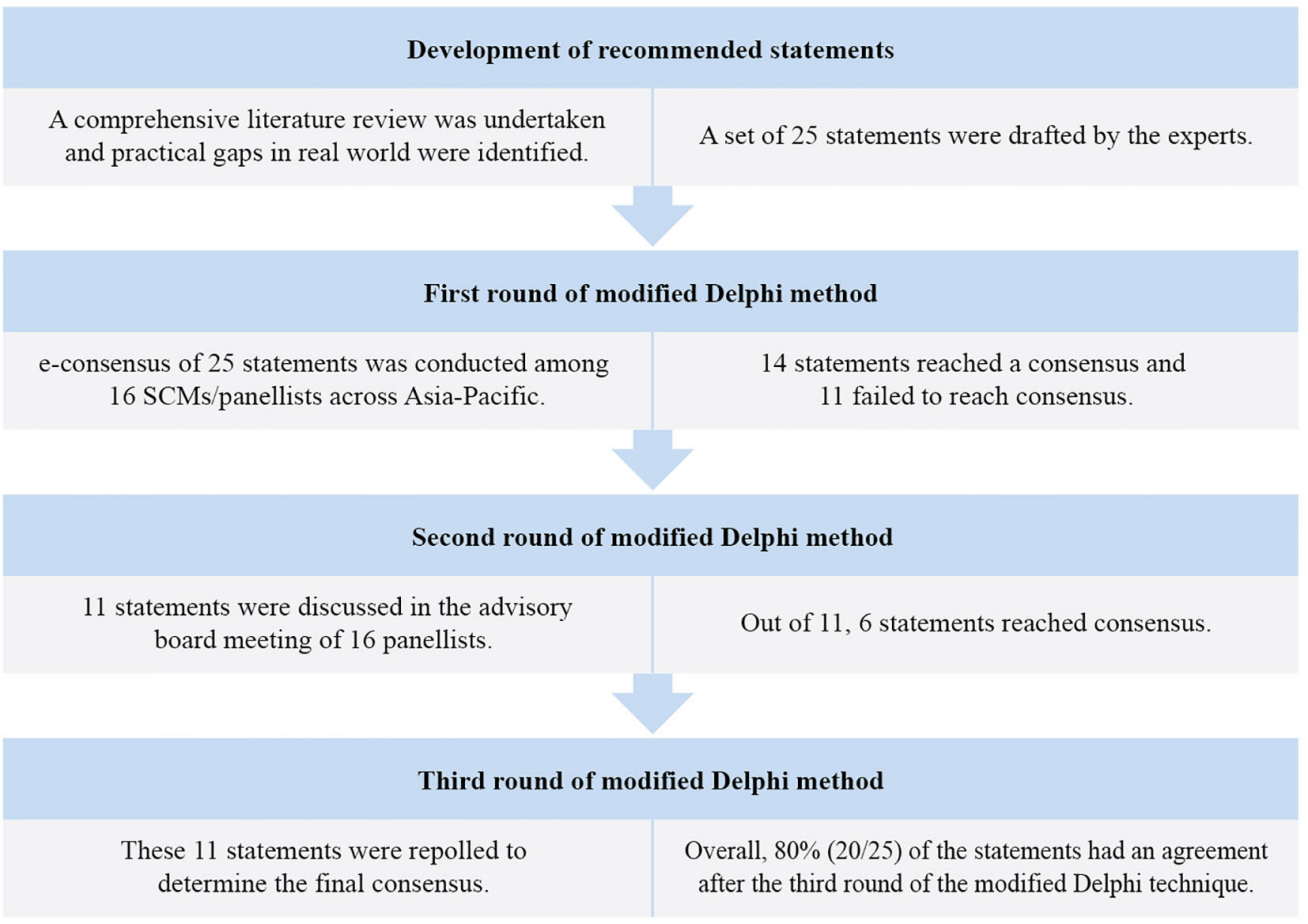
Modified Delphi method for the development of consensus.

Of the 25 statements in the first round of the e-consensus survey, 14 reached a consensus, whereas 11 did not reach a consensus. The 11 statements that did not reach initial consensus were discussed and revised during the scientific advisory board meeting (**Figure 2**) of which six statements reached consensus. The statements were graded for quality of evidence using the Oxford level of evidence. About 15 statements (60%) were of high quality, one statement (4%) was of moderate quality, and nine statements (36%) were of very low quality (**Supplementary Table S3**). The level of consensus among the SCMs was high for 20 statements (80%), moderate for three statements (12%), and low for two statements (8%). Results are summarised under the domains outlined in **Tables 1**–**5**.

**Table 1:** Surgical interventions in *BRCA* germline pathogenic variants in *HER2*-negative eBC.

Sl. no.	Consensus statements	Agree (%)	Level of consensus
1	It is ideal to know the *BRCA* germline pathogenic variants status of an *HER2*-negative eBC patient before definitive surgery as the information can impact surgical intervention decisions.	81·3	High
2	For women with BC who are known to carry a *BRCA* germline likely/pathogenic variants scheduled for therapeutic mastectomy on the affected side, CRRM should be discussed.	87·5	High
3	RRM is the most effective and known preventive measure for BC in *BRCA* germline pathogenic variant carriers, with a 90%–95% risk reduction rate.	81·3	High
4	In patients with *BRCA* germline pathogenic variants who develop primary BC, breast-conserving surgery is not contraindicated; however, ipsilateral therapeutic mastectomy and CRRM should be discussed considering the increased risk of ipsilateral and contralateral new primary BC compared with that in patients with sporadic cancers.	87·5	High

BC, Breast cancer; CRRM, Contralateral risk-reducing mastectomy; eBC, Early breast cancer*;* g, Germline*; HER2,* Human epidermal growth factor receptor 2; RRM, Risk-reducing mastectomy.

Level of consensus: High ( ≥75%), moderate (55%–74%), low (<55%)

**Table 2:** Treatment sequencing in eTNBC patients carrying *BRCA* germline pathogenic variants.

Sl. no.	Consensus statements	Agree (%)	Level of consensus
5	There is robust clinical evidence to demonstrate that PARPi significantly reduces the risk of disease recurrence and provides a clinically meaningful extension of overall survival in *BRCA* germline pathogenic variant carriers with high-risk *HER2*-negative eTNBC.	87·5	High
6	Once *BRCA* germline likely/pathogenic variant is detected in high-risk eTNBC, PARPi should be considered as part of the treatment plan.	93·8	High
7	Before initiating treatment with adjuvant PARPi in *BRCA* germline pathogenic variant carriers with high-risk eTNBC, patients should have received at least 4–8 cycles of prior neoadjuvant CT in the absence of CT intolerance.	87·5	High
8	The incorporation of platinum agents in neoadjuvant CT increases pCR rates and may be considered in *BRCA* germline pathogenic variant carriers with eTNBC.	81·3	High
9	There is limited evidence on the use of platinum derivatives in the adjuvant setting, and its use in the adjuvant setting remains controversial*. (N=14)	85·7	High
10	In eTNBC patients who received neoadjuvant IO containing CT, GeparNuevo suggested that similar outcomes can be achieved by neoadjuvant IO without extending IO use in an adjuvant setting after surgery*. (N=13)	76·9	High
11	Adjuvant PARPi is recommended in *BRCA* germline pathogenic variant carriers with eTNBC who do not achieve pCR following neoadjuvant therapy.	87·5	High
12	In eTNBC patients with *BRCA* germline pathogenic variants, who do not achieve pCR following neoadjuvant CT + IO, although there is limited evidence on efficacy and safety, individual consideration can be given in selected patients to administer adjuvant PARPi concurrently with adjuvant IO or to administer sequential adjuvant IO and PARPi*. (N=13)	84·6	High
13	Although there is no current evidence, adjuvant PARPi may be considered in eTNBC patients carrying *BRCA* germline pathogenic variants who have achieved pCR from neoadjuvant therapy, based on the risk of disease recurrence from the initial clinical stage*. (N=13)	46·2	Low
14	*BRCA* germline pathogenic variant carriers with high-risk eTNBC who have not received neoadjuvant CT (with or without IO) should receive adjuvant CT followed by adjuvant PARPi.	68·8	Moderate
15	For *BRCA* germline pathogenic variant carriers with eTNBC who fail to achieve pCR after neoadjuvant CT only (without IO), preference should be given to PARPi over capecitabine as adjuvant therapy.	75	High
16	There is growing evidence that patients with BC with basal-like histology have suboptimal outcomes with capecitabine in the adjuvant setting. Basal-like histology is more prominent in *BRCA* carriers.	56·3	Moderate

BC, Breast cancer; CT, Chemotherapy; eTNBC, Early triple-negative breast cancer; *HER2,* Human epidermal growth factor receptor 2; IO, Immunotherapy; PARPi, Poly (ADP-ribose) polymerase inhibitor; pCR, Pathological complete response.

*Statement nos. 10, 12, and 13 included responses from 13 experts, and Statement no. 9 included responses from 14 experts.

Level of consensus: High ( ≥75%), moderate (55%–74%), low (<55%)

**Table 3:** Treatment sequencing in *BRCA* germline pathogenic variants in HR*-*positive/*HER2*-negative eBC.

Sl. no.	Consensus statements	Agree (%)	Level of consensus
17	There is robust clinical evidence to demonstrate that PARPi significantly reduces the risk of disease recurrence and provides a clinically meaningful extension of overall survival in *BRCA* germline pathogenic variant carriers with high-risk early HR*-*positive *HER2*-negative BC.	75	High
18	For *BRCA* germline pathogenic variant carriers with high-risk HR*-*positive *HER2-*negative eBC, PARPi should be considered as part of the treatment plan.	87·4	High
19	For *BRCA* germline pathogenic variant carriers with high-risk HR*-*positive *HER2-*negative eBC, ET + PARPi may be preferred over ET + CDK4/6i as escalated adjuvant treatment*. (N=13)	76·9	High
20	In *BRCA* germline pathogenic variant carriers with HR*-*positive *HER2-*negative eBC at very high risk of recurrence (e.g. T >5 cm, grade 3, Ki67 >30%), sequential escalated adjuvant therapy can be considered with ET + PARPi (1 year) followed by ET + CDK4/6i (2 years)*. (N=13)	38·5	Low

BC, Breast cancer; CDK4/6i, Cyclin-dependent kinase 4/6 inhibitor; eBC, Early breast cancer*;* ET, Endocrine therapy; *HER2,* Human epidermal growth factor receptor 2; HR, Hormone receptor; PARPi, Poly (ADP-ribose) polymerase inhibitor.

*Statement nos. 19 and 20 included responses from 13 experts.

Level of consensus: High ( ≥75%), moderate (55%–74%), low (<55%)

**Table 4:** *HER2*-negative metastatic BC carrying *BRCA* germline pathogenic variant.

Sl. no.	Consensus statements	Agree (%)	Level of consensus
21	If resources permit, GC/GT should be offered to all *HER2*-negative metastatic BC patients to guide therapeutic decisions at the diagnosis of metastatic disease.	93·7	High
22	There is strong clinical evidence that PARPi delays disease progression in *BRCA* germline pathogenic variant carriers with metastatic *HER2*-negative BC who have previously been treated with CT in the neoadjuvant, adjuvant, or metastatic setting.	87·5	High
23	PARPi should be given preference as upfront therapy over CT ± IO in *BRCA* germline pathogenic variant carriers with *de novo* or recurrent metastatic TNBC*. (N=13)	30·8	Low

BC, Breast cancer; CT, Chemotherapy; g, Germline; GC, Genetic counselling; GT, Genetic testing*; HER2,* Human epidermal growth factor receptor 2; IO, Immunotherapy; PARPi, Poly (ADP-ribose) polymerase inhibitor; TNBC, Triple-negative breast cancer.

*Statement no. 23 included responses from 13 experts.

Level of consensus: High ( ≥75%), moderate (55%–74%), low (<55%)

**Table 5:** Miscellaneous.

Sl. no.	Consensus statements	Agree (%)	Level of consensus
24	There is no current evidence to use adjuvant PARPi combined with anti-HER2–directed therapies in *BRCA* germline pathogenic variant carriers with *HER2-*positive eBC*. (N=13)	92·3	High
25	Limited evidence suggests suboptimal outcomes with ET ± CDK4/6i in HR-positive *HER2*-negative patients carrying *BRCA* germline pathogenic variants or pathogenic variants in other HRR pathway genes*. (N=13)	92·3	High

CDK4/6i, Cyclin-dependent kinase 4/6 inhibitor; eBC, Early breast cancer*;* ET, Endocrine therapy; g, Germline*; HER2,* Human epidermal growth factor receptor 2; HRR, Homologous recombination repair; PARPi, Poly (ADP-ribose) polymerase inhibitor.

*Statement nos. 24 and 25 included responses from 13 experts.

Level of consensus: High ( ≥75%), moderate (55%–74%), low (<55%)

### 3.1 Surgical interventions in *BRCA* germline pathogenic variants in *HER2*-negative eBC

The SCMs reached a high consensus for the statement on determining *BRCA* status of *HER2*-negative eBC patients before surgery to influence surgical decisions; discussing contralateral risk-reducing mastectomy (CRRM) for women with *BRCA* pathogenic variants scheduled for therapeutic mastectomy; risk-reducing mastectomy (RRM) being an effective preventive measure for *BRCA* variant carriers; and considering breast-conserving surgery for *BRCA* carriers with primary BC (Statements 1 to 4, **Table 1**).

### 3.2 Treatment sequencing in eTNBC patients carrying *BRCA* germline pathogenic variants

A high consensus was reached by the SCMs for PARPi reducing disease recurrence and improving overall survival (OS) in high-risk *HER2*-negative eTNBC with *BRCA* germline pathogenic variant; considering PARPi in the treatment plan after detection of *BRCA* germline likely/pathogenic variant; considering prerequisites related to prior neoadjuvant chemotherapy before starting PARPi; incorporating platinum agents in neoadjuvant chemotherapy to increase pCR rates; achieving outcomes with neoadjuvant IO therapy alone, which is similar to outcomes without extending IO in the adjuvant setting (as per GeparNeuvo study); recommending adjuvant PARPi in patients who do not achieve pCR following neoadjuvant therapy; administering PARPi with IO based on individual consideration; and preferring PARPi over capecitabine as adjuvant therapy who do not achieve pCR after neoadjuvant chemotherapy (without IO). A moderate consensus was reached by SCMs for providing adjuvant chemotherapy followed by PARPi in patients who did not receive neoadjuvant chemotherapy, having suboptimal outcomes with capecitabine in the adjuvant setting in patients with basal-like histology. A low consensus was reached by SCMs for considering adjuvant PARPi for eTNBC patients who achieved pCR from neoadjuvant therapy (based on initial recurrence risk) (Statements 5 to 16, **Table 2**).

### 3.3 Treatment sequencing in *BRCA* germline pathogenic variants in HR-positive/*HER2*-negative eBC

A high consensus was reached by the SCMs on the effectiveness of PARPi in reducing recurrence and extending survival in *BRCA* carriers with high-risk HR-positive/*HER2*-negative BC; including PARPi in the treatment plan for high-risk *BRCA* carriers with HR-positive/*HER2*-negative eBC; and preferring endocrine therapy (ET) + PARPi over ET + cyclin-dependent kinase 4/6 inhibitor (CDK4/6i) for high-risk HR-positive/*HER2*-negative eBC. A low consensus was reached by the SCMs on considering sequential therapy with ET + PARPi followed by ET + CDK4/6i for very high-risk *BRCA* carriers with HR-positive/*HER2*-negative eBC (Statements 17 to 20, **Table 3**).

### 3.4 *HER2*-negative metastatic BC carrying *BRCA* germline pathogenic variant

A high consensus was reached by the SCMs for offering genetic counselling (GC)/genetic testing (GT) to all *HER2*-negative metastatic BC patients if resources permit and having a delayed progression after the use of PARPi in *BRCA* carriers with metastatic *HER2*-negative BC. A low consensus was reached by the SCMs for preferring PARPi over chemotherapy ± IO for *BRCA* carriers with metastatic TNBC (Statements 21 to 23, **Table 4**).

### 3.5 Other *BRCA* germline pathogenic variant subtypes of BC

A high consensus was reached on having no evidence supporting the adjuvant PARPi with anti-*HER2* therapies for *HER2*-positive eBC in *BRCA* carriers and having suboptimal outcomes with ET ± CDK4/6i in HR-positive/*HER2*-negative *BRCA* carriers (based on limited evidence) (Statements 24 to 25, **Table 5**).

### 3.6 HCP survey

Differences were observed between real-world practices and SCM recommendations for scenarios such as discussing CRRM with the patients as a part of standard practice (HCPs [41·8%] *vs*. SCMs [87·5%]) and considering PARPi administration concurrently or sequentially with IO as a treatment option for eTNBC patients carrying *BRCA* germline pathogenic variants who do not achieve pCR following neoadjuvant CT + IO (HCPs [50%] *vs*. SCMs [84·6%]).

Additionally, differences between real-world practices and SCM recommendations were also observed for the use of adjuvant PARPi in patients with *BRCA* germline pathogenic variants in eTNBC who have achieved pCR from neoadjuvant therapy (HCPs [14·2%] *vs*. SCMs [46·2%]). The preference for ET + PARPi over ET + cyclin-dependent kinase 4/6 inhibitors (CDK4/6i) as escalated adjuvant treatment for *BRCA* pathogenic variants with high-risk HR+*/HER2*-negative eBC differed (HCPs [41%] *vs*. SCMs [76·9%]). Considering PARPi treatment for patients with metastatic *BRCA* germline pathogenic variants TNBC who have been previously treated with CT also differed (HCPs [66·4%] *vs*. SCMs [87·5%]). The detailed agreement, disagreement, and abstain percentages are presented in **Supplementary Table S3**, and the results of the real-world survey are available in **Supplementary Table S4**. The details of the HCPs (country-wise and specialty-wise) are available in **Supplementary Figures S1** and **Supplementary Figure S2**. The results of the real-world questionnaire are discussed and compared wherever applicable in the discussion section. These consensus results are further discussed under the same domains with supporting literature in the subsequent section.

## 4 Discussion

### 4.1 Surgical interventions in *BRCA* germline pathogenic variants in *HER2*-negative eBC

A significant change in the surgical management of primary BC has been the transition towards breast conservation techniques, which began >30 years ago (12). The optimal surgical treatment for operable BC in *BRCA1/2*m pathogenic variant carriers depends on several factors and remains debated (**Table 1**).

The panel recommended (81·3%) identifying the *BRCA* germline pathogenic variants status of a patient with *HER2*-negative eBC before definitive surgery as it can impact surgical intervention decisions (Statement no. 1) (13, 14). Contemplating the significantly higher risk for ipsilateral and contralateral new primary BC development, it was proposed that as standard practice, ipsilateral therapeutic mastectomy and CRRM must be discussed with *BRCA* germline pathogenic variants carriers (Statement no. 4). This statement is also supported by the National Comprehensive Cancer Network^®^ (NCCN) guidelines (15) and other literature (2, 13, 16, 17). A difference was observed in real-world practice as only 41·8% of the HCPs follow this as standard practice in the Asia-Pacific region. Risk-reducing salpingo-oophorectomy (RRSO) proves to be an efficacious strategy for mitigating BC and ovarian cancer risk in females harbouring *BRCA1/2* germline pathogenic variants, particularly in those of a younger age group. Implementing RRSO within the initial 5-year period after surgery could offer substantial advantages to individuals with *BRCA1/2* germline pathogenic variants (18). According to the NCCN guidelines, women with a known *BRCA1*/2 pathogenic or likely pathogenic variant should undergo RRSO, specifically between the ages of 35 and 40 years for those with a *BRCA1* pathogenic or likely pathogenic variant and between the ages of 40 and 45 years for those with a *BRCA2* pathogenic or likely pathogenic variant (19). A meta-analysis reported an increase in overall survival in patients with *BRCA1/2* pathogenic variant carriers (when analysed both the variants together) who underwent RRSO compared to those who did not undergo RRSO. Additionally, undergoing RRSO led to an improvement in the quality of life (QoL) in terms of perception of the risk of developing BC, compared to those who did not have the RRSO procedure (20). However, in Southeast Asian countries, the practice of HCPs may likely be hindered based on the priorities of women at different stages of life. Many women are concerned about how the post-surgical changes might affect their motherhood responsibilities. The importance of their husband’s support and approval, along with concerns about how the surgery might impact their emotional well-being and sexuality, further complicates the decision (21). Nevertheless, it should be noted that a large proportion of women experience menopausal symptoms or clinical manifestations of oestrogen deficiency during breast cancer treatment or after completing therapy. These effects may include accelerated bone loss, an increased incidence of osteoporotic fractures, cardiovascular diseases, vulvovaginal atrophy, and sexual dysfunction. Given these potential adverse outcomes, it is crucial to balance the benefits of cancer risk reduction with the long-term physiological and QoL implications. Strategies such as bone health management optimizing Vitamin D levels, cardiovascular risk reduction by maintaining healthy lifestyle, and interventions to address sexual and menopausal health should be integrated into the care plan to minimize these adverse effects while ensuring effective cancer control (22).

### 4.2 Treatment sequencing in eTNBC carrying *BRCA* germline pathogenic variants

Triple-negative BC is the most aggressive form of BC, affecting 15%–20% of all cases. Recently, PARPis have been approved for treating patients with *BRCA*1/2 germline pathogenic variants BC (23, 24). The statements related to treatment sequencing in patients with *BRCA* germline pathogenic variants in eTNBC are presented in **Table 2**.

#### 4.2.1 Platinum agents in neoadjuvant and adjuvant settings for eBC

Anthracyclines and taxanes are typically the foundations of neoadjuvant CT for patients with TNBC. Using platinum and taxane-based CT in the neoadjuvant and adjuvant setting may improve the survival of patients with eTNBC by achieving a high pCR (24). Although clinical guidelines and consensus papers provide contrasting viewpoints on the use of platinum agents in patients with TNBC, consideration for including platinum agents in neoadjuvant CT for patients with *BRCA* germline pathogenic variants in eTNBC reached a high consensus (81·3%) among the panel (Statement no. 8).There is an ongoing debate regarding the benefit of adding a platinum agent to standard neoadjuvant CT. The BrighTNess study, with a follow-up period of 4·5 years, demonstrated that adding carboplatin to neoadjuvant CT (paclitaxel) improved pCR and event-free survival (EFS) without increasing the incidence of myelodysplastic syndrome or acute myeloid leukaemia (25). Likewise, a network meta-analysis including 35 RCTs also reported results favouring the addition of platinum agents to neoadjuvant CT for treating TNBC. However, it was highlighted that these agents when combined with other targeted therapies to accomplish higher benefits are associated with higher toxicity (26). The use of neoadjuvant platinum is supported by the European Society of Medical Oncology (ESMO) Clinical Practice Guidelines, St. Gallen/Vienna 2023 consensus discussion, and the American Society of Clinical Oncology (ASCO). The NCCN reports recommend that the benefits are more pronounced in younger or premenopausal patients, with substantial improvements in EFS and OS (12, 24, 27–29).

There was a high consensus (85·7%) among the panel that there is limited evidence on the usage of platinum derivatives in adjuvant settings, which is in concordance with another consensus paper (Statement no. 9) (30). The NCCN and ESMO guidelines also discourage adjuvant treatment with platinum agents (12, 24, 31). The panel discussed that only about 25% of the population of the OlympiA trial received platinum as neoadjuvant/adjuvant therapy and currently, more patients are treated with neoadjuvant platinum in the real world because of data from the KEYNOTE-522 trials and the BrighTNess study. The KEYNOTE-522 trial used a standard neoadjuvant CT backbone with anthracycline, taxane, and platinum in high-risk eTNBC cases (32). A few panellists opined that using platinum agents in the neoadjuvant setting justifies their use in the adjuvant setting.

#### 4.2.2 IO in the adjuvant setting for eTNBC

The panel reached a consensus (76·9%) in alignment with the GeparNuevo study, which suggested that similar outcomes can be achieved with neoadjuvant IO alone without extending IO into the adjuvant setting after surgery in patients with eTNBC (Statement no. 10). Although the differences in outcomes in the GeparNeuvo study were not statistically significant, the long-term outcome data from the G trial indicated that patients receiving neoadjuvant durvalumab (*vs*. placebo) demonstrated higher rates of 3-year invasive disease-free, distant disease-free, and OS even without additional IO therapy in the adjuvant setting (33). This observation underscores the notion that the primary therapeutic impact of IO therapy may be achieved through early intervention. According to the GeparNeuvo study, administering IO (durvalumab) alongside CT before surgery triggers a systemic immunological response that effectively controls or eliminates distant tumour cells and micrometastases. This early intervention initiates significant alterations in intratumoural tumour-infiltrating lymphocytes (iTILs), which play a pivotal role in modulating the immune system’s response against cancer. The changes also aid in predicting the success of the treatment in achieving pCR. These observations suggest that the primary benefit of IO may be maximised during the neoadjuvant phase, where richer tumour antigens exist to prime the immune system before surgical resection. Consequently, there may be no additional benefit from continuing IO into the adjuvant phase after surgery (34). In contrast, the phase III KEYNOTE-522 trial, which included long-term follow-up data, demonstrated a benefit in EFS when IO therapy was incorporated both in the neoadjuvant and adjuvant settings. At the 36-month follow-up, the EFS was higher in the group receiving CT + pembrolizumab (84·5%, 95% confidence interval [CI]=81·7%–86·9%) which incorporated IO in neoadjuvant and adjuvant setting compared with the groups receiving CT alone (76·8%, 95% CI=72·2%–80·7%) (34). At 60 months, the EFS rate remained consistently higher in the pembrolizumab + CT group (81·3%, 95% CI=78·4%–83·9%) compared to the CT alone group (72%, 95% CI=67·5%-76·5%) (35). Serious treatment-related adverse events occurred in 34·1% of the patients in the study group and 20·1% of patients in the control group. Based on the findings of the KEYNOTE-522 study, the United States Food and Drug Administration (U.S. FDA) approved the use of both, neoadjuvant pembrolizumab + chemotherapy and the adjuvant pembrolizumab monotherapy in high-risk eTNBC (34). However, the St. Gallen/Vienna 2023 consensus panel had a divided opinion on whether adjuvant pembrolizumab should be administered in patients with pCR, Only 59% of the panel agreed that it should be used, while 32% disagreed, and 9% did not vote (27). While the current guidelines recommend completing both neoadjuvant and adjuvant pembrolizumab for eTNBC based on the KEYNOTE-522 trial showing long-term EFS benefit, the trial does not differentiate the specific contributions of the IO in neoadjuvant *vs*. adjuvant settings to this benefit. This lack of clarity leaves uncertainty about the necessity of continuing pembrolizumab after surgery. Thus, while the trial supports using IO in both settings, it does not provide definitive evidence that the adjuvant phase offers a substantial additional benefit. This ambiguity is significant in contexts where the high cost of treatment poses a significant barrier. In many Asian countries where patients pay out of pocket, the high cost of treatment is a major barrier. Therefore, rational allocation of health resources is crucial. Given this context of limitations in the KEYNOTE-522 trial and considering the cost constraints, using neoadjuvant pembrolizumab alone is likely still beneficial compared to not using pembrolizumab at all. Thus, many experts in the present study agreed that neoadjuvant IO without adjuvant IO is acceptable.

#### 4.2.3 PARPi in an adjuvant setting for eTNBC

The panel agreed that PARPi significantly reduces the risk of disease recurrence, provides a clinically meaningful extension of OS in *BRCA* germline pathogenic variants carriers with high-risk eTNBC, and should be considered part of the clinical practice treatment plan (Statement nos. 5 and 6). The outcomes of several clinical trials highlight the importance of using PARPi among patients with early-stage BC and those with advanced disease. The panel recommended that patients should receive at least 4–8 cycles of neoadjuvant CT in the absence of CT intolerance before initiating treatment with adjuvant PARPi in *BRCA* germline pathogenic variant carriers with high-risk eTNBC (Statement no. 7). Administering 6–8 cycles of neoadjuvant CT to optimise the pCR rate has become the standard treatment in clinical practice (36). The SCM concluded that high-quality evidence from clinical trials needed to be adopted in routine practice by HCPs. Additionally, real-world studies are required to understand better the epidemiology of *BRCA* germline pathogenic variants and clinicopathological differences in BC between Western and Asian countries.

The panel reached a moderate consensus (68·8%) for the statement ‘*BRCA* germline pathogenic variant carriers with high-risk eTNBC who have not received neoadjuvant CT (with or without IO) should receive adjuvant CT followed by adjuvant PARPi’ (Statement no. 14). The statement is supported by the OlympiA trial, where all eligible patients had received neoadjuvant or adjuvant CT (6). The real-world survey conducted in this paper showed that 64·2% of the HCPs would consider adjuvant CT followed by PARPi for the same scenario.

The panel reached a high consensus (84·6%) that treatment decisions should be individualised when considering the concurrent or sequential administration of adjuvant PARPi *vs*. adjuvant IO in patients with *BRCA* germline pathogenic variants in eTNBC who do not achieve pCR following neoadjuvant CT + IO therapy. The SCMs agreed that there are currently no safety and efficacy data available for the concurrent administration of PARPi and IO in eBC cases, although safety data from the combination are available in the metastatic settings (i.e. from the TOPACIO11, MEDIOLA, KEYLYNK-007, and KEYLYNK-013 trials) (Statement no. 12). To overcome resistance and optimise the advantageous outcomes of ICIs, innovative approaches emphasising combinations of ICIs with other therapies have been under investigation. PARPis represent a promising class of agents capable of synergising effectively in ICI-based combined therapies (37). Indeed, the Dana-Farber group favoured concurrent administration of both agents if adequately tolerated (38). In the real-world survey, only 50% of HCPs considered administering adjuvant PARPi concurrently or sequentially with IO. The gap between SCM recommendations and real-world practices may be because it is more difficult for real-world adoption due to compounded out-of-pocket costs and a lack of direct clinical trial evidence to ascertain superiority over the standard approach.

The panellists agreed that there is no evidence to support the use of adjuvant PARPi in patients with eTNBC carrying *BRCA* germline pathogenic variants who have achieved pCR from neoadjuvant therapy based on the risk of disease recurrence from the initial clinical stage of the tumour. The panel’s opinions were split almost equally among the panellists who agreed (46·2%), disagreed (30·7%), and abstained (23·1%) on not supporting the use of adjuvant PARPi in patients with eTNBC carrying *BRCA* germline pathogenic variants who have achieved pCR from neoadjuvant therapy based on the risk of disease recurrence from the initial clinical stage of the tumour (Statement no. 13). There is similarly a low consensus on this among HCPs in the real world with only 14·2% of the surveyed HCPs opting for this treatment approach (**Supplementary Table S4**, Q4).

The SCMs reached a consensus in prioritising PARPi over capecitabine as adjuvant therapy in patients with *BRCA* germline pathogenic variants in eTNBC who fail to achieve pCR after neoadjuvant therapy. The panellists acknowledged that there is no head-to-head data comparing the efficacies of capecitabine and olaparib as adjuvant therapies (Statement no. 15). The CREATE-X, KEYNOTE-522, and OlympiA trials have changed the treatment paradigms for eTNBC cases. In the phase III CREATE-X study, adjuvant capecitabine increased invasive disease-free survival (DFS) and OS in patients with eTNBC with residual disease after neoadjuvant CT (39). However, the administration of capecitabine was not allowed in the OlympiA and KEYNOTE-522 studies. The literature indicates that olaparib can be cautiously preferred over capecitabine as adjuvant therapy in *BRCA1/2* germline pathogenic variant TNBC cases as the PARPi directly targets the deoxyribonucleic acid (DNA) damage repair pathway implicated in this tumour subset. Additionally, during data analysis, the CREATE-X, SYSUCC-001, and CBCSG010 trials did not consider patients’ *BRCA1/2* germline pathogenic variant status. *Post hoc* analysis of the FinXX trial demonstrated that combining capecitabine with adjuvant CT may be more beneficial in non-*BRCA-*like tumours than in *BRCA*-like tumours (40).

The majority of patients with *BRCA*1m in eTNBC have basal-like tumours, which are sensitive to regimens containing standard DNA-damaging agents, such as anthracyclines and cyclophosphamide; non-basal eTNBCs, however, seem particularly sensitive to the addition of capecitabine to standard (neo)adjuvant CT (41). Correlative information from the GEICAM-CIBOMA study revealed that patients with non-basal*-*like tumours derived significant improvements in invasive DFS and OS with capecitabine treatment compared with those with basal-like tumours (42). Similarly, in the ECOG-ACRIN EA1131 study, non-basal subtypes appeared to benefit more from capecitabine therapy than platinum-based CT (43). However, only 56·3% of SCMs agreed that patients with basal-like histology have suboptimal outcomes with capecitabine in the adjuvant setting (Statement no. 16).

### 4.3 Treatment sequencing in *BRCA* germline pathogenic variants in HR*-*positive/*HER2*-negative eBC

The treatment of HR-positive/*HER2-*negative BC varies by stage and tumour characteristics; however, ET remains the mainstay of treatment in both early and advanced stages of this BC type. Chemotherapy is administered as needed based on the biology and extent of the disease. Targeted therapies, such as CDK4/6i or PARPis for patients with a *BRCA1/2* germline pathogenic variant, are recommended in patients with high-risk disease. With the availability of new therapy options, it is also critical to determine the best sequence of treatments to optimise clinical benefit while avoiding harm (44). The statements related to treatment sequencing in patients with *BRCA* germline pathogenic variants in HR*-*positive/*HER2*-negative eBC are presented in **Table 3**.

The SCMs reached a high consensus on the use of PARPi in *BRCA* germline pathogenic variant carriers with high-risk early HR-positive *HER2*-negative BC (Statement no. 17) (6, 9). A subgroup analysis of the OlympiAD trial involving Asian patients with *BRCA* germline pathogenic variants and *HER2*-negative metastatic BC reported that the patients treated with PARPi (olaparib) had a longer median progression-free survival than those receiving chemotherapy (5·7 months *vs*. 4·2 months; hazard ratio [HR] = 0·53) (45). The use of PARPi has been shown to improve the quality of life (QoL) for patients, evidenced by an increase in Quality of Life Questionnaire Core 30 (QLQ-C30) scores (encompassing physical, role, emotional, cognitive, and social functioning) from baseline to various time points. In contrast, the standard therapy group experienced a decline in their QLQ-C30 scores (46). However, PARPi are associated with a range of side effects such as anaemia, fatigue, nausea, and vomiting. Additionally, serious adverse events can occur, such as myelodysplastic syndrome/acute myeloid leukaemia (MDS/AML), pneumonitis, and venous thromboembolism, thus managing side effects associated with PARPi is crucial for optimum QoL (47). The panel preferred ET + PARPi over ET + CDK4/6i as escalated adjuvant treatment in *BRCA* germline pathogenic variants high-risk HR*-*positive *HER2*-negative eBC cases. However, the panellists agreed that while there was no direct evidence for this preference, there was a scientific rationale. To their knowledge, there is no data for a head-to-head comparison of the efficacies of the two treatments; however, considering the OS benefits of olaparib in patients with *BRCA* germline pathogenic variants, with efficacy seen in the HR+ subset, 76·9% of SCMs preferred the administration of ET + PARPi over ET + CDK4/6i in these patients (Statement no. 19) (9). A correlative study from Memorial Sloan Kettering Cancer Center (MSKCC) also suggested that CDK4/6i does not offer any significant benefits to *BRCA*2 germline pathogenic variant carriers with metastatic BC (mBC) due to CDK4/6i resistance (38). In comparison, in the real-world survey, only 41% of HCPs preferred ET + PARPi over ET + CDK4/6i (**Supplementary Table S4**, Q6). It is important to note that there is a lack of head-to-head comparison between adjuvant ET+PARPi *vs*. ET + CDK4/6i in HR+/*HER2*- eBC carrying *BRCA* pathogenic variants. HCPs are, therefore, often led to prescribe a reimbursed or lower cost treatment option.

For *BRCA* germline pathogenic variant carriers with HR*-*positive *HER2*-negative eBC at very high risk of recurrence, there is very low consensus among the panel (38·5%) that sequential escalated adjuvant therapy can be considered with ET + PARPi (for one year) followed by ET + CDK4/6i (for two years). The panel implies that there is no evidence available in the literature to support this treatment approach (Statement no. 20). The administration of adjuvant olaparib followed by abemaciclib may be considered in selected high-risk patients after careful discussions and weighing the risk–benefit ratio for the individual patient (27).

### 4.4 *HER2*-negative metastatic BC carrying *BRCA* germline pathogenic variant

The phase III OlympiAD trial confirmed the effectiveness of olaparib in individuals with hereditary *BRCA* germline pathogenic variants and *HER2*-negative mBC, with a statistically significant improvement in progression-free survival compared with the physician’s choice of CT treatment (**Table 4**) (46). The median final OS was 19.3 months with olaparib and 17.1 months with the physician’s choice of CT, which was comparable between the two groups (48).

About 61.5% experts agreed that there is a lack of evidence for using PARPi as an upfront therapy over CT ± IO in *BRCA* germline pathogenic variant carriers with *de novo* or relapsed metastatic disease. A moderate consensus of 30·8% was reached for using PARPi as upfront therapy. One of the panellists preferred PARPi after considering the outcomes of patients treated with olaparib in the first-line metastatic setting in the OlympiAD trial and suggested that earlier use of PARPi improves OS. Another panellist favoured PARPi over CT ± IO after comparing the adverse effects of both therapies. However, many experts voiced their opinions that if PARPi is given as first-line therapy, patients with programmed death-ligand 1 (PD-L1)-positive tumours could miss the chance of receiving IO as IO + CT is approved only as first-line treatment (Statement no. 23).

### 4.5 Other *BRCA* germline pathogenic variant subtypes of BC

Currently, the evidence lacks to support the use of adjuvant PARPi in combination with anti-*HER2*–directed therapies for *BRCA* germline pathogenic variant carriers with *HER2*-positive eBC (49). While PARPi have demonstrated efficacy in *BRCA*-mutated, *HER2*-negative BC, their role in *HER2*-positive disease remains unclear because the clinical trials for the PARPi did not include *HER2*-positive BCs. Since *HER2*-driven tumours rely on distinct survival pathways (50) it may reduce their dependence on PARP-mediated DNA repair and, in turn, limit the therapeutic efficacy of this combination. Given the lack of clinical evidence supporting the benefit, a high level of consensus was reached regarding lack of evidence on PARPi in combination with anti-*HER2*–directed therapies (Statement no. 24). About, 92·3% of the SCMs agreed that there is limited evidence that treatment with ET ± CDK4/6i offers suboptimal outcomes in patients who are HR*-*positive/*HER2*-negative carrying *BRCA* germline pathogenic variants in other homologous recombination repair pathway genes. Nonetheless, the panellists acknowledged the growing evidence and noted that most data are from the metastatic setting (Statement no. 25).

The limitation of this study is the low response rate of the HCPs to the survey questionnaire. However, it should be noted that HCPs who responded were from ten different Asia-Pacific nations including Australia, India, Indonesia, Malaysia, Philippines, South Korea, Taiwan, Singapore, Thailand, and Vietnam. These include both developed and developing countries, ensuring that the findings are relevant and applicable across different healthcare systems, cultures, and patient populations within the region. Another limitation is that, although experts were well-renowned experts in the Asia-Pacific region for the management of BC who had more than ten years of experience and had published articles in peer-reviewed journals, there were still some discrepancies among experts due to differing ethnic backgrounds and country-specific accessibility to resources, related to socioeconomic status rather than scientific background, which may have influenced the findings.

## 5 Conclusion

**Figure 3:**
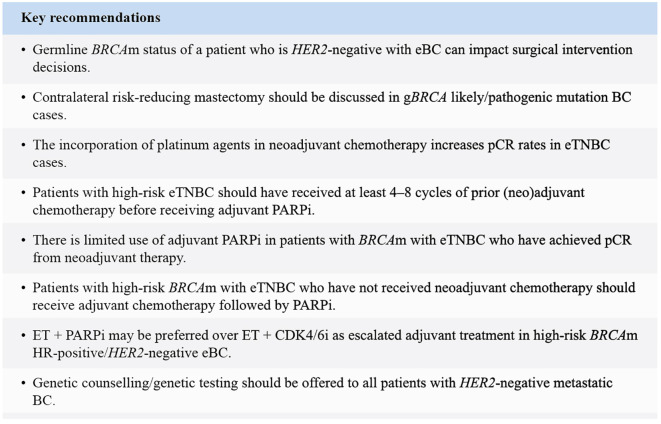
Key recommendations. BC, Breast cancer; CDK4/6i, Cyclin-dependent kinase 4/6 inhibitor; eBC, Early breast cancer; ET, Endocrine therapy; eTNBC, Early triple-negative breast cancer; g, Germline; *HER2,* Human epidermal growth factor receptor 2; HR, Hormone receptor; m, Mutation; PARPi, Poly (ADP-ribose) polymerase inhibitor; pCR, Pathological complete response.

This paper provides practical guidance on the surgical management and systemic therapies available for patients with *BRCA* germline pathogenic variants in *HER2*-negative BC in the Asia-Pacific region. Testing for *BRCA* germline pathogenic variants should be expanded to include all young patients with BC (<45 years of age) as this subpopulation in the Asia-Pacific region has the highest probability of harbouring *BRCA* germline pathogenic variants. However, given that *BRCA* carriers can also present beyond 45 years of age and *BRCA* status impacts treatment decisions, broader testing criteria, potentially up to 65 years of age should be considered, aligning with international guidelines. A high consensus was reached in 80% of the statements, while some areas of controversy persisted, and no consensus could be reached for those statements. Patients with *BRCA* germline pathogenic variants were recommended to undergo genetic testing before surgery as it can impact surgical intervention decisions and further systemic treatment. These patients can also benefit from prophylactic surgical interventions. The use of neoadjuvant platinum agents in CT increases the pCR rate. Adjuvant PARPi is preferred over capecitabine in patients with *BRCA* germline pathogenic variants with eTNBC and over CDK4/6i as escalated treatment in patients who are HR*-*positive *HER2*-negative. It is anticipated that this consensus paper and the expert recommendations addressing real-world practical scenarios included here will enhance patient care and service quality across the Asia-Pacific region. The key recommendations of the experts are presented in **Figure 3**.
