# Correction: Part II: consensus statements and expert recommendations for BRCA-associated breast cancer in the Asia-Pacific region: clinical management

**DOI:** 10.3389/fonc.2026.1807980

**Published:** 2026-03-09

**Authors:** Yeon Hee Park, Soo Chin Lee, Christian F. Singer, Judith Balmaña, Rebecca Alexandra Dent, Veronique Kiak-Mien Tan, Nadia Ayu Mulansari, Mastura Md. Yusof, Frances Victoria F. Que, Yen-Shen Lu, Napa Parinyanitikul, Cam Phuong Pham, Nur Aishah Taib, Sun-Young Kong, Yoland Antill, Hee Jeong Kim

**Affiliations:** 1 Division of Haematology-Oncology, Department of Medicine, Samsung Medical Centre, Sungkyunkwan University School of Medicine, Seoul, Republic of Korea; 2 Department of Haematology-Oncology, National University Cancer Institute, Singapore, Singapore; 3 Department of Obstetrics and Gynaecology, Comprehensive Cancer Centre, Medical University of Vienna, Vienna, Austria; 4 Department of Medical Oncology, University Hospital Campus Vall Hebron, Barcelona, Spain; 5 Division of Medical Oncology, National Cancer Centre Singapore, Singapore, Singapore; 6 Department of Breast Surgery, Division of Surgery and Surgical Oncology, National Cancer Centre Singapore, Singapore, Singapore; 7 Haematology-Medical Oncology Division, Internal Medicine Department, Cipto Mangunkusumo National General Hospital/Universitas Indonesia, Jakarta, Indonesia; 8 Picaso Cancer Centre, Hospital Picaso, Petaling Jaya, Selangor, Malaysia; 9 Department of Internal Medicine and Oncology, St Luke’s Medical Center, Quezon City and Global City, Metro Manila, Philippines; 10 Department of Oncology, National Taiwan University Hospital, Taipei, Taiwan; 11 Medical Oncology Unit, King Chulalongkorn Memorial Hospital, Bangkok, Thailand; 12 The Nuclear Medicine and Oncology Center, Bachmai Hospital, Hanoi, Vietnam; 13 Nuclear Medicine Department, Hanoi Medical University, Hanoi, Vietnam; 14 Oncology and Nuclear Medicine Department, University of Medicine and Pharmacy, Vietnam National University, Hanoi, Vietnam; 15 Department of Surgery, Faculty of Medicine, University Malaya, UM Cancer Research Institute, Kuala Lumpur, Malaysia; 16 Department of Laboratory Medicine and Genetic Counselling Clinic, National Cancer Center, Goyang, Gyeonggi-do, Republic of Korea; 17 Familial Cancer Centre, Royal Melbourne Hospital, Melbourne, VIC, Australia; 18 Faculty of Medicine and Health Sciences, Monash University, Melbourne, VIC, Australia; 19 Division of Breast Surgery, Department of Surgery, Asan Medical Center, University of Ulsan College of Medicine, Seoul, Republic of Korea

**Keywords:** BRCA germline pathogenic variants, early breast cancer, HER2, PARP inhibitors, triple-negative breast cancer

In the published article, there was a mistake in the text and citation in Section 4.4. *“HER2-negative metastatic BC carrying BRCA germline pathogenic variant”*. In paragraph 1 of the section 4.4, the following text and citation was incorrect “The phase III OlympiAD trial confirmed the effectiveness of olaparib in individuals with hereditary BRCA germline pathogenic variants and HER2-negative mBC, with a statistically significant improvement in progression-free survival and OS compared with the physician’s choice of CT treatment **(Table 4) (48)**. The 4-year OS in the olaparib group was 89·8% compared with 86·4% in the placebo group **(9)**.”

The correct text and citation should be:

“The phase III OlympiAD trial confirmed the effectiveness of olaparib in individuals with hereditary BRCA germline pathogenic variants and HER2-negative mBC, with a statistically significant improvement in progression-free survival compared with the physician’s choice of CT treatment **(Table 4) (46)**. The median final OS was 19.3 months with olaparib and 17.1 months with the physician’s choice of CT, which was comparable between the two groups **(48)**.”

The original version of this article has been updated.

