# Supplementary material for: Part II: consensus statements and expert recommendations for BRCA-associated breast cancer in the Asia-Pacific region: clinical management
Source: Front Oncol. 2025 Jun 23;15:1507840. doi: 10.3389/fonc.2025.1507840 (PMC12230081; doi:10.3389/fonc.2025.1507840)
Supplement: Supplementary file 1 [file DataSheet1.doc]

**Supplementary Material**

**Supplementary Table S1.** Expert panel for the consensus

| **Name of the member** | **Specialty** | **Place** |
| --- | --- | --- |
| Prof. Lee Soo Chin (Co-chair) | Medical Oncologist/Geneticist | Singapore |
| Prof. Christian Singer (Co-chair) | Medical Oncologist | Austria |
| Prof. Judith Balmana | Medical Oncologist | Spain |
| A/Prof. Rebecca Dent | Medical Oncologist | Singapore |
| Dr. Veronique Tan | Breast Surgeon | Singapore |
| A/Prof. Yoland Antill | Medical Oncologist/Geneticist | Australia |
| Dr. Nadia Ayu Mulansari | Medical Oncologist | Indonesia |
| Dr. Mastura Md Yusof | Medical Oncologist | Malaysia |
| Dr. Frances Victoria Que | Medical Oncologist | Philippines |
| Prof. Yeon Hee Park | Medical Oncologist | S. Korea |
| Prof. Yen Shen Lu | Medical Oncologist | Taiwan |
| Assoc. Prof. Napa Parinyanitikul | Medical Oncologist | Thailand |
| Assoc. Prof. Pham Cam Phuong | Medical Oncologist | Vietnam |
| Prof. Nur Aishah Taib | Breast Surgeon/Geneticist | Malaysia |
| Prof. Sun Young Kong | Geneticist | S. Korea |
| Prof. Hee Jeong Kim | Breast Surgeon | S. Korea |

**Supplementary Table S2.** Search strategy used for literature search

Search limit: Up to 31 October 2022

| **Search no.** | **Search string** |
| --- | --- |
| **PubMed** | |
| **#1** | ("Breast Neoplasms"[MeSH Terms] OR breast cancer[MeSH Terms] OR "Breast Cancer"[Title/Abstract] OR "Breast Carcinoma"[Title/Abstract] OR “early breast cancer” OR "Malignant Breast Tumor"[Title/Abstract] OR "Invasive Breast Cancer"[Title/Abstract] OR "Non-Invasive Breast Cancer"[Title/Abstract] OR "Ductal Carcinoma In Situ"[Title/Abstract] OR "Lobular Carcinoma In Situ"[Title/Abstract] OR "Triple-Negative Breast Cancer"[Title/Abstract] OR “human epidermal growth factor receptor 2” [Title/Abstract] OR "HER2-negative Breast Cancer"[Title/Abstract] OR “HR-positive”[All Fields] OR "Metastatic Breast Cancer"[Title/Abstract] OR "BRCA1 Protein"[MeSH Terms] OR "Genes, BRCA1"[MeSH Terms] OR "Genes, BRCA2"[MeSH Terms] OR "breast cancer susceptibility"[Title/Abstract] OR "hereditary breast cancer"[Title/Abstract] |
| **#2** | "DNA testing"[Title/Abstract] OR "genomic testing"[Title/Abstract] OR "mutation screening"[Title/Abstract]) OR ("Diagnosis"[MeSH Terms] OR “genomic stability"[Title/Abstract] OR "cancer risk assessment”[Title/Abstract] OR "diagnosis"[Title/Abstract] OR "diagnostic assessment"[Title/Abstract] OR "early detection"[Title/Abstract] OR "early diagnosis"[Title/Abstract] OR "Genetic Testing"[MeSH Terms] OR "Genetic Counseling"[MeSH Terms] |
| **#3** | "Clinical Management"[Title/Abstract] OR "Patient Care Management"[MeSH Terms] OR "treatment sequencing"[Title/Abstract] OR "patient care management"[Title/Abstract] OR "treatment planning"[Title/Abstract] OR "clinical guidelines"[Title/Abstract] OR "management guidelines"[Title/Abstract] OR "treatment guidelines"[Title/Abstract] OR "therapeutic strategies"[Title/Abstract] OR "clinical decision making"[Title/Abstract] OR "multidisciplinary care"[Title/Abstract] OR "personalized medicine"[Title/Abstract] OR "targeted therapy"[Title/Abstract] OR "chemotherapy"[Title/Abstract] OR "radiation therapy"[Title/Abstract] OR "surgical options"[Title/Abstract] OR "hormonal therapy"[Title/Abstract] OR "immunotherapy"[Title/Abstract] OR "palliative care"[Title/Abstract] |
| **#4** | "Risk Assessment"[MeSH Terms] OR "risk assessment"[Title/Abstract] OR "risk analysis"[Title/Abstract] OR "risk evaluation"[Title/Abstract] OR "risk factors"[Title/Abstract] OR "cancer risk factors"[Title/Abstract] OR "breast cancer risk"[Title/Abstract] OR "predictive modeling"[Title/Abstract] OR "prognostic factors"[Title/Abstract] OR "cancer prediction models"[Title/Abstract] OR "cancer prognosis"[Title/Abstract] OR "survival analysis"[MeSH Terms] OR "survival rate"[Title/Abstract] OR "genetic predisposition to disease"[MeSH Terms] OR "genetic risk"[Title/Abstract] OR "family history"[Title/Abstract] OR "hereditary risk"[Title/Abstract] OR "lifestyle risk factors"[Title/Abstract] OR "environmental risk factors"[Title/Abstract] |
| **#5** | "Recurrence"[MeSH Terms] OR "cancer recurrence"[Title/Abstract] OR "breast cancer recurrence"[Title/Abstract] OR "local recurrence"[Title/Abstract] OR "regional recurrence"[Title/Abstract] OR "distant recurrence"[Title/Abstract] OR "relapse"[Title/Abstract] OR "cancer relapse"[Title/Abstract] OR "breast cancer relapse"[Title/Abstract] OR "recurrent breast cancer"[Title/Abstract] OR "tumor recurrence, local"[MeSH Terms] OR "second primary neoplasms"[MeSH Terms] OR "secondary cancer"[Title/Abstract] OR "metachronous neoplasms"[Title/Abstract] OR "second primary cancer"[Title/Abstract] |
| **#6** | “Asia-Pacific”[All Fields] OR “Afghanistan”[MeSH Terms] OR “Australia”[MeSH Terms] OR “Bangladesh”[MeSH Terms] OR “Bhutan”[MeSH Terms] OR “Burma”[All Fileds] OR “Brunei”[All Fields] OR “Cambodia”[MeSH Terms] OR “China”[MeSH Terms] OR “Cook Islands”[MeSH Terms] OR “Federated States of Micronesia”[MeSH Terms] OR “Fiji”[MeSH Terms] OR “India”[MeSH Terms] OR “Indonesia”[MeSH Terms] OR “Japan”[ MeSH Terms] OR “Kiribati”[MeSH Terms] OR “Laos”[MeSH Terms] OR “Malaysia”[MeSH Terms] OR “Maldives”[MeSH Terms] OR “Marshall Islands”[MeSH Terms] OR “Mongolia”[MeSH Terms] OR “Nepal”[MeSH Terms] OR “New Caledonia”[MeSH Terms] OR “New Zealand”[MeSH Terms] OR “Niue”[MeSH Terms] OR “North Korea”[All Fields] OR “Pakistan”[MeSH Terms] OR “Palau”[MeSH Terms] OR “Papua New Guinea”[MeSH Terms] OR “Philippines”[MeSH Terms] OR “Singapore”[MeSH Terms] OR “Solomon Islands”[MeSH Terms] OR “South Korea”[MeSH Terms] OR “Sri Lanka”[MeSH Terms] OR “Taiwan”[MeSH Terms] OR “Thailand”[MeSH Terms] OR “Timor-Leste” [MeSH Terms] OR “Tonga”[MeSH Terms] OR “Tuvalu”[MeSH Terms] OR “Vanuatu”[MeSH Terms] OR “Vietnam”[MeSH Terms] |
| **#7**  **(Diagnosis and Management)** | #1 AND #2 AND #3 AND #6 |
| **#8**  **(Risk assessment and recurrence)** | #1 AND #4 AND #5 AND #6 |
| **Cochrane** | |
| **#1** | ([mh "Breast Neoplasms"] OR [mh "breast cancer"] OR "Breast Cancer":ti,ab OR "Breast Carcinoma":ti,ab OR "early breast cancer" OR "Malignant Breast Tumor":ti,ab OR "Invasive Breast Cancer":ti,ab OR "Non-Invasive Breast Cancer":ti,ab OR "Ductal Carcinoma In Situ":ti,ab OR "Lobular Carcinoma In Situ":ti,ab OR "Triple-Negative Breast Cancer":ti,ab OR "human epidermal growth factor receptor 2":ti,ab OR "HER2-negative Breast Cancer":ti,ab OR HR-positive OR "Metastatic Breast Cancer":ti,ab OR [mh "BRCA1 Protein"] OR [mh "Genes, BRCA1"] OR [mh "Genes, BRCA2"] OR "breast cancer susceptibility":ti,ab OR "hereditary breast cancer":ti,ab) |
| **#2** | ("DNA testing":ti,ab OR "genomic testing":ti,ab OR "mutation screening":ti,ab) OR ([mh Diagnosis] OR "genomic stability":ti,ab OR "cancer risk assessment":ti,ab OR diagnosis:ti,ab OR "diagnostic assessment":ti,ab OR "early detection":ti,ab OR "early diagnosis":ti,ab OR [mh "Genetic Testing"] OR [mh "Genetic Counseling"]) |
| **#3** | ("Clinical Management":ti,ab OR [mh "Patient Care Management"] OR "treatment sequencing":ti,ab OR "patient care management":ti,ab OR "treatment planning":ti,ab OR "clinical guidelines":ti,ab OR "management guidelines":ti,ab OR "treatment guidelines":ti,ab OR "therapeutic strategies":ti,ab OR "clinical decision making":ti,ab OR "multidisciplinary care":ti,ab OR "personalized medicine":ti,ab OR "targeted therapy":ti,ab OR chemotherapy:ti,ab OR "radiation therapy":ti,ab OR "surgical options":ti,ab OR "hormonal therapy":ti,ab OR immunotherapy:ti,ab OR "palliative care":ti,ab) |
| **#4** | ([mh "Risk Assessment"] OR "risk assessment":ti,ab OR "risk analysis":ti,ab OR "risk evaluation":ti,ab OR "risk factors":ti,ab OR "cancer risk factors":ti,ab OR "breast cancer risk":ti,ab OR "predictive modeling":ti,ab OR "prognostic factors":ti,ab OR "cancer prediction models":ti,ab OR "cancer prognosis":ti,ab OR [mh "survival analysis"] OR "survival rate":ti,ab OR [mh "genetic predisposition to disease"] OR "genetic risk":ti,ab OR "family history":ti,ab OR "hereditary risk":ti,ab OR "lifestyle risk factors":ti,ab OR "environmental risk factors":ti,ab) |
| **#5** | ([mh Recurrence] OR "cancer recurrence":ti,ab OR "breast cancer recurrence":ti,ab OR "local recurrence":ti,ab OR "regional recurrence":ti,ab OR "distant recurrence":ti,ab OR relapse:ti,ab OR "cancer relapse":ti,ab OR "breast cancer relapse":ti,ab OR "recurrent breast cancer":ti,ab OR [mh "tumor recurrence, local"] OR [mh "second primary neoplasms"] OR "secondary cancer":ti,ab OR "metachronous neoplasms":ti,ab OR "second primary cancer":ti,ab) |
| **#6** | (Asia-Pacific:ti,ab OR Afghanistan:ti,ab OR Australia:ti,ab OR Bangladesh:ti,ab OR Bhutan:ti,ab OR "Burma":ti,ab OR Brunei:ti,ab OR Cambodia:ti,ab OR China:ti,ab OR "Cook Islands":ti,ab OR "Federated States of Micronesia":ti,ab OR Fiji:ti,ab OR India:ti,ab OR Indonesia:ti,ab OR "Japan":ti,ab OR Kiribati:ti,ab OR Laos:ti,ab OR Malaysia:ti,ab OR Maldives:ti,ab OR "Marshall Islands":ti,ab OR Mongolia:ti,ab OR Nepal:ti,ab OR "New Caledonia":ti,ab OR "New Zealand":ti,ab OR Niue:ti,ab OR "North Korea":ti,ab OR Pakistan:ti,ab OR Palau:ti,ab OR "Papua New Guinea":ti,ab OR Philippines:ti,ab OR Singapore:ti,ab OR "Solomon Islands":ti,ab OR "South Korea":ti,ab OR "Sri Lanka":ti,ab OR Taiwan:ti,ab OR Thailand:ti,ab OR Timor-Leste:ti,ab OR Tonga:ti,ab OR Tuvalu:ti,ab OR Vanuatu:ti,ab OR Vietnam:ti,ab) |
| **#7**  **(Diagnosis and Management)** | #1 AND #2 AND #3 AND #6 |
| **#8**  **(Risk assessment and recurrence)** | #1 AND #4 AND #5 AND #6 |
| **Embase** | |
| #1 | ('Breast Neoplasms'/exp OR 'breast cancer'/exp OR 'Breast Cancer':ti,ab OR 'Breast Carcinoma':ti,ab OR 'early breast cancer' OR 'Malignant Breast Tumor':ti,ab OR 'Invasive Breast Cancer':ti,ab OR 'Non-Invasive Breast Cancer':ti,ab OR 'Ductal Carcinoma In Situ':ti,ab OR 'Lobular Carcinoma In Situ':ti,ab OR 'Triple-Negative Breast Cancer':ti,ab OR 'human epidermal growth factor receptor 2':ti,ab OR 'HER2-negative Breast Cancer':ti,ab OR HR-positive OR 'Metastatic Breast Cancer':ti,ab OR 'BRCA1 Protein'/exp OR 'Genes, BRCA1'/exp OR 'Genes, BRCA2'/exp OR 'breast cancer susceptibility':ti,ab OR 'hereditary breast cancer':ti,ab) |
| #2 | ('DNA testing':ti,ab OR 'genomic testing':ti,ab OR 'mutation screening':ti,ab) OR (Diagnosis/exp OR 'genomic stability':ti,ab OR 'cancer risk assessment':ti,ab OR diagnosis:ti,ab OR 'diagnostic assessment':ti,ab OR 'early detection':ti,ab OR 'early diagnosis':ti,ab OR 'Genetic Testing'/exp OR 'Genetic Counseling'/exp) |
| #3 | ('Clinical Management':ti,ab OR 'Patient Care Management'/exp OR 'treatment sequencing':ti,ab OR 'patient care management':ti,ab OR 'treatment planning':ti,ab OR 'clinical guidelines':ti,ab OR 'management guidelines':ti,ab OR 'treatment guidelines':ti,ab OR 'therapeutic strategies':ti,ab OR 'clinical decision making':ti,ab OR 'multidisciplinary care':ti,ab OR 'personalized medicine':ti,ab OR 'targeted therapy':ti,ab OR chemotherapy:ti,ab OR 'radiation therapy':ti,ab OR 'surgical options':ti,ab OR 'hormonal therapy':ti,ab OR immunotherapy:ti,ab OR 'palliative care':ti,ab) |
| #4 | ('Risk Assessment'/exp OR 'risk assessment':ti,ab OR 'risk analysis':ti,ab OR 'risk evaluation':ti,ab OR 'risk factors':ti,ab OR 'cancer risk factors':ti,ab OR 'breast cancer risk':ti,ab OR 'predictive modeling':ti,ab OR 'prognostic factors':ti,ab OR 'cancer prediction models':ti,ab OR 'cancer prognosis':ti,ab OR 'survival analysis'/exp OR 'survival rate':ti,ab OR 'genetic predisposition to disease'/exp OR 'genetic risk':ti,ab OR 'family history':ti,ab OR 'hereditary risk':ti,ab OR 'lifestyle risk factors':ti,ab OR 'environmental risk factors':ti,ab) |
| #5 | (Recurrence/exp OR 'cancer recurrence':ti,ab OR 'breast cancer recurrence':ti,ab OR 'local recurrence':ti,ab OR 'regional recurrence':ti,ab OR 'distant recurrence':ti,ab OR relapse:ti,ab OR 'cancer relapse':ti,ab OR 'breast cancer relapse':ti,ab OR 'recurrent breast cancer':ti,ab OR 'tumor recurrence, local'/exp OR 'second primary neoplasms'/exp OR 'secondary cancer':ti,ab OR 'metachronous neoplasms':ti,ab OR 'second primary cancer':ti,ab) |
| #6 | (Asia-Pacific OR Afghanistan/exp OR Australia/exp OR Bangladesh/exp OR Bhutan/exp OR ‘Burma[All fields]’ OR Brunei OR Cambodia/exp OR China/exp OR ‘Cook Islands’/exp OR ‘Federated States of Micronesia’/exp OR Fiji/exp OR India/exp OR Indonesia/exp OR ‘Japan[ MeSH Terms]’ OR Kiribati/exp OR Laos/exp OR Malaysia/exp OR Maldives/exp OR ‘Marshall Islands’/exp OR Mongolia/exp OR Nepal/exp OR ‘New Caledonia’/exp OR ‘New Zealand’/exp OR Niue/exp OR ‘North Korea’ OR Pakistan/exp OR Palau/exp OR ‘Papua New Guinea’/exp OR Philippines/exp OR Singapore/exp OR ‘Solomon Islands’/exp OR ‘South Korea’/exp OR ‘Sri Lanka’/exp OR Taiwan/exp OR Thailand/exp OR Timor-Leste/exp OR Tonga/exp OR Tuvalu/exp OR Vanuatu/exp OR Vietnam/exp) |
| **#7**  **(Diagnosis and Management)** | #1 AND #2 AND #3 AND #6 |
| **#8**  **(Risk assessment and recurrence)** | #1 AND #4 AND #5 AND #6 |

**Supplementary Table S3.** Consensus statements

| **Sl. No.** | **Consensus statement** | **Level of evidence, Grade** | **Agree**  **(%)** | **Disagree**  **(%)** | **Abstain**  **(%)** | **Level of consensus** |
| --- | --- | --- | --- | --- | --- | --- |
|  | **Section 3: Surgical interventions in *BRCA* germline pathogenic variants in *HER2*-negative eBC** | | | | | |
| 1 | It is ideal to know the *BRCA* germline pathogenic variants status of a *HER2*-negative eBC patient before definitive surgery as the information can impact surgical intervention decisions. (N=16) | 1a, High | 13 (81∙3) | 1 (6∙2) | 2 (12∙5) | High |
| 2 | For women with BC who are known to carry a *BRCA* germline likely /pathogenic variants scheduled for therapeutic mastectomy on the affected side, CRRM should be discussed. (N=16) | 1a, High | 14 (87∙5) | 0 | 2 (12∙5) | High |
| 3 | RRM is the most effective and known preventive measure for BC in *BRCA* germline pathogenic variant carriers, with a 90%–95% risk reduction rate. (N=16) | 1a, High | 13 (81∙3) | 2 (12∙5) | 1 (6∙2) | High |
| 4 | In patients *BRCA* germline pathogenic variants who develop primary BC, breast-conserving surgery is not contraindicated; however, ipsilateral therapeutic mastectomy and CRRM should be discussed considering the increased risk of ipsilateral and contralateral new primary BC compared with patients with sporadic cancers. (N=16) | 1a, High | 14 (87∙5) | 0 | 2 (12∙5) | High |
|  | **Section 4a: Treatment sequencing in eTNBC patients carrying *BRCA* germline pathogenic variants** | | | | | |
| 5 | There is robust clinical evidence to demonstrate that PARPi significantly reduces the risk of disease recurrence and provides a clinically meaningful extension of overall survival in *BRCA* germline pathogenic variant carriers with high‑risk *HER2*-negative eTNBC. (N=16) | 1b, High | 14 (87∙5) | 0 | 2 (12∙5) | High |
| 6 | Once *BRCA* germline likely/pathogenic variant is detected in high-risk eTNBC, PARPi should be considered as part of the treatment plan. (N=16) | 1b, High | 15 (93∙8) | 0 | 1 (6∙2) | High |
| 7 | Before initiating treatment with adjuvant PARPi in *BRCA* germline pathogenic variant carriers with high-risk eTNBC, patients should have received at least 4–8 cycles of prior neoadjuvant CT in the absence of CT intolerance. (N=16) | 1a, High | 14 (87∙5) | 0 | 2 (12∙5) | High |
| 8 | The incorporation of platinum agents in neoadjuvant CT increases pCR rates and may be considered in *BRCA* germline pathogenic variant carriers with eTNBC. (N=16) | 1a, High | 13 (81∙3) | 1 (6∙2) | 2 (12∙5) | High |
| 9 | There is limited evidence on the use of platinum derivatives in the adjuvant setting, and its use in the adjuvant setting remains controversial*. (N=14) | 1a, High | 12 (85∙7) | 2 (14∙3) | 0 | High |
| 10 | In eTNBC patients who received neoadjuvant IO containing CT, GeparNeuvo suggested that similar outcomes can be achieved by neoadjuvant IO without extending IO use in an adjuvant setting after surgery*. (N=13) | 1b, Moderate | 10 (76∙9) | 1 (7∙7) | 2 (15∙4) | High |
| 11 | Adjuvant PARPi is recommended in *BRCA* germline pathogenic variant carriers with eTNBC who do not achieve pCR following neoadjuvant therapy. (N=16) | 1a, High | 14 (87∙5) | 0 | 2 (12∙5) | High |
| 12 | In eTNBC patients with *BRCA* germline pathogenic variants, who do not achieve pCR following neoadjuvant CT+IO, although there is limited evidence on efficacy and safety, individual consideration can be given in selected patients to administer adjuvant PARPi concurrently with adjuvant IO or to administer sequential adjuvant IO and PARPi*. (N=13) | 1a, High | 11 (84∙6) | 1 (7∙7) | 1 (7∙7) | High |
| 13 | Although there is no current evidence, adjuvant PARPi may be considered in eTNBC patients carrying *BRCA* germline pathogenic variants who have achieved pCR from neoadjuvant therapy, based on the risk of disease recurrence from the initial clinical stage*. (N=13) | 5, Very low | 6 (46∙2) | 4 (30∙7) | 3 (23∙1) | Low |
| 14 | *BRCA* germline pathogenic variant carriers with high-risk eTNBC who have not received neoadjuvant CT (with or without IO) should receive adjuvant CT followed by adjuvant PARPi. (N=16) | 5, Very low | 11 (68∙8) | 0 | 5 (31∙2) | Moderate |
| 15 | For *BRCA* germline pathogenic variant carriers with eTNBC who fail to achieve pCR after neoadjuvant CT only (without IO), preference should be given to PARPi over capecitabine as adjuvant therapy. (N=16) | 5, Very low | 12 (75) | 1 (6∙2) | 3 (18∙8) | High |
| 16 | There is growing evidence that patients with BC with basal-like histology have suboptimal outcomes with capecitabine in the adjuvant setting. Basal‑like histology is more prominent in *BRCA* carriers. (N=16) | 5, Very low | 9 (56∙3) | 1 (6∙2) | 6 (37∙5) | Moderate |
|  | **Section 4b: Treatment sequencing in *BRCA*** **germline pathogenic variants in HR-positive *HER2*-negative eBC** | | | | | |
| 17 | There is robust clinical evidence to demonstrate that PARPi significantly reduces the risk of disease recurrence and provided a clinically meaningful extension of overall survival in *BRCA* germline pathogenic variant carriers with high‑risk early HR*-*positive *HER2*-negative BC. (N=16) | 1b, High | 12 (75) | 2 (12∙5) | 2 (12∙5) | High |
| 18 | For *BRCA* germline pathogenic variant carriers with high-risk HR*-*positive *HER2-*negative eBC, PARPi should be considered as part of the treatment plan. (N=16) | 5, Very low | 14 (87∙6) | 1 (6∙2) | 1 (6∙2) | High |
| 19 | For *BRCA* germline pathogenic variant carriers with high‑risk HR*-*positive *HER2-*negative eBC, ET + PARPi may be preferred over ET + CDK4/6i as escalated adjuvant treatment*. (N=13) | 5, Very low | 10 (76∙9) | 0 | 3 (23∙1) | High |
| 20 | In *BRCA* germline pathogenic variant carriers with HR*-*positive *HER2-*negative eBC at very high risk of recurrence (e.g. T >5 cm, grade 3, Ki67 >30%), sequential escalated adjuvant therapy can be considered with ET+PARPi (1 year) followed by ET+CDK4/6i (2 years)*. (N=13) | 5, Very low | 5 (38∙5) | 3 (23) | 5 (38∙5) | Low |
|  | **Section 4c: *HER2*-negative metastatic BC carrying *BRCA* germline pathogenic variant** | | | | | |
| 21 | If resources permit, GC/GT should be offered to all *HER2*-negative metastatic BC patients to guide therapeutic decisions at the diagnosis of metastatic disease. (N=16) | 1a, High | 93∙7 | 6∙3 | 0 | High |
| 22 | There is strong clinical evidence that PARPi delays disease progression in *BRCA* germline pathogenic variant carriers with metastatic *HER2*-negative BC who have previously been treated with CT in the neoadjuvant, adjuvant, or metastatic setting. (N=16) | 1b, High | 14 (87∙5) | 0 | 12∙5 | High |
| 23 | PARPi should be given preference as upfront therapy over CT±IO in *BRCA* germline pathogenic variant carriers with *de novo* or recurrent metastatic TNBC*. (N=13) | 1a, High | 4 (30∙8) | 8 (61∙5) | 1 (7∙7) | Low |
|  | **Section 4d: Other *BRCA* germline pathogenic variant** **subtypes of BC** | | | | | |
| 24 | There is no current evidence to use adjuvant PARPi combined with  anti-*HER2*–directed therapies in *BRCA* germline pathogenic variant carriers with *HER2-*positive eBC*. (N=13) | 5, Very low | 12 (92∙3) | 0 | 1 (7∙7) | High |
|  | **Miscellaneous** | | | | | |
| 25 | There is limited evidence suggesting suboptimal outcomes with ET+/-CDK4/6i in HR‑positive *HER2*‑negative patients carrying *BRCA* germline pathogenic variant or pathogenic variants in other HRR pathway genes*. (N=13) | 5, Very low | 12 (92∙3) | 0 | 1 (7∙7) | High |

BC: Breast cancer; CDK4/6i: Cyclin-dependent kinase 4/6 inhibitor; CRRM: Contralateral risk-reducing mastectomy; CT: Chemotherapy; eBC: Early breast cancer*;* ET: Endocrine therapy; eTNBC: Early triple-negative breast cancer; GC: Genetic counselling; GT: Genetic testing*; HER-2:* Human epidermal growth factor receptor 2; HR: Hormone receptor; HRR: Homologous recombination repair; IO: Immunotherapy; PARPi: Poly ADP‑ribose polymerase inhibitor; pCR: Pathological complete response; TNBC: Triple-negative breast cancer.

*Statement No. 9 included responses from 14 experts, and statement Nos. 10, 12, 13, 19, 20, 23, 24, and 25 included responses from 13 experts as the remaining experts voted as “not related to the field of expertise.”

Colour coding represents:

|  | High consensus |
| --- | --- |
|  | Moderate consensus |
|  | Low consensus |

**Supplementary Table S4:** Real-world survey response

| **Sl. No.** | **Consensus statements from which the questionnaire was developed** | **Question** | **Response of HCPs**  **(N)** | **Response of HCPs**  **(%)** | **Agreement of SCMs to the statements (%)** | **Inference** |
| --- | --- | --- | --- | --- | --- | --- |
| **1.** |  | **In my country, breast cancer patients with *BRCA* germline pathogenic variants are usually considered for further surgical options, such as contralateral RRM.** | | |  |  |
| a | In patients with *BRCA* germline pathogenic variants who develop primary BC, breast-conserving surgery is not contraindicated; however, ipsilateral therapeutic mastectomy and CRRM should be discussed considering the increased risk of ipsilateral and contralateral new primary BC compared with that in patients with sporadic cancers. | Yes | 67 | 50∙0 | 87∙5 | A significant gap observed between real-world practice and recommendations of SCMs |
| b |  | No | 7 | 5∙2 |  |  |
| c |  | Not a standard practice | 56 | 41∙8 |  |  |
| d |  | I do not know | 2 | 1∙5 |  |  |
| e |  | Abstain | 2 | 1∙5 |  |  |
| **2.** |  | **In my current practice, a majority of eTNBC patients would receive?** | | |  |  |
| a | Before initiating treatment with adjuvant PARPi in *BRCA* germline pathogenic variant carriers with high-risk eTNBC, patients should have received at least 4–8 cycles of prior neoadjuvant CT in the absence of CT intolerance. | Neoadjuvant CT | 89 | 66∙4 | 87∙5 | Real-world practice concurs with the recommendations of SCMs |
| b |  | Neoadjuvant CT+ IO | 27 | 20∙1 |  |  |
| c |  | No neoadjuvant CT | 9 | 6∙7 |  |  |
| d |  | Abstain | 9 | 6∙7 |  |  |
| **3.** |  | **Which is your preferred adjuvant treatment option for eTNBC patients with *BRCA* germline pathogenic variants who do not achieve pCR following neoadjuvant CT+ IO?** | | |  |  |
| a |  | PARPi monotherapy | 33 | 24∙6 |  |  |
| b | In eTNBC patients with *BRCA* germline pathogenic variants, who do not achieve pCR following neoadjuvant CT + IO, although there is limited evidence on efficacy and safety, individual consideration can be given in selected patients to administer adjuvant PARPi concurrently with adjuvant IO or to administer sequential adjuvant IO and PARPi. | Adjuvant PARPi concurrently with adjuvant IO | 41 | 30∙6 | 84∙6 | A significant gap observed between real-world practice and recommendations of SCMs |
| c |  | Sequential adjuvant IO followed by adjuvant PARPi | 23 | 17∙2 |  |  |
| d |  | IO monotherapy | 5 | 3∙7 |  |  |
| e |  | Abstain | 24 | 17∙9 |  |  |
| f |  | Others | 15 | 6∙0 |  |  |
| **4.** |  | **My preferred adjuvant treatment option for eTNBC patients with *BRCA*** **germline pathogenic variants who achieved pCR from neoadjuvant therapy of CT+/-IO would be** | | |  |  |
| a | Although there is no current evidence, adjuvant PARPi may be considered in eTNBC patients carrying *BRCA* germline pathogenic variants who have achieved pCR from neoadjuvant therapy, based on the risk of disease recurrence from the initial clinical stage | Adjuvant PARPi | 19 | 14∙2 | 46∙2 | A significant gap observed between real-world practice and recommendations of SCMs |
| b |  | Adjuvant CT | 7 | 5∙2 |  |  |
| c |  | Adjuvant IO | 32 | 23∙9 |  |  |
| d |  | Adjuvant IO followed by adjuvant PARPi | 11 | 8∙2 |  |  |
| e |  | Adjuvant IO + PARPi | 7 | 5∙2 |  |  |
| f |  | No adjuvant treatment | 41 | 30∙6 |  |  |
| g |  | Abstain | 17 | 12∙7 |  |  |
| **5.** |  | **Which is your preferred adjuvant treatment option for high-risk eTNBC patients with *BRCA*** **germline pathogenic variants who have not received neoadjuvant CT?** | | |  |  |
| a | *BRCA* germline pathogenic variant carriers with high-risk eTNBC who have not received neoadjuvant CT (with or without IO) should receive adjuvant CT followed by adjuvant PARPi. | CT followed by PARPi | 86 | 64∙2 | 68∙8 | Real-world practice concurs with the recommendations of SCMs |
| b |  | Capecitabine | 6 | 4∙5 |  |  |
| c |  | Platinum-based CT | 26 | 19∙4 |  |  |
| d |  | Abstain | 12 | 9∙0 |  |  |
| e |  | Others | 4 | 3∙0 |  |  |
| **6.** |  | **Which is your preferred adjuvant treatment option for *BRCA*** **germline pathogenic variants with high-risk HR+*/HER2*-negative eBC?** | | |  |  |
| a |  | CDK4/6i followed by PARPi | 10 | 7∙5 |  |  |
| b | For *BRCA* germline pathogenic variant carriers with high-risk HR-positive *HER2*-negative eBC, ET + PARPi may be preferred over ET + CDK4/6i as escalated adjuvant treatment. | ET + PARPi | 55 | 41∙0 | 76∙9 | A significant gap observed between real-world practice and recommendations of SCMs |
| c |  | PARPi followed by CDK4/6i | 14 | 10∙4 |  |  |
| d |  | ET + CDK4/6i | 33 | 24∙6 |  |  |
| e |  | Abstain | 16 | 11∙9 |  |  |
| f |  | Others | 8 | 4∙5 |  |  |
| **7.** |  | **For *BRCA* germline pathogenic variant carriers with metastatic TNBC, who have previously received CT, my next treatment option would be?** | | |  |  |
| a |  | CT | 5 | 3∙7 |  |  |
| b |  | CT + IO | 25 | 18∙7 |  |  |
| c | For *BRCA* germline pathogenic variant carriers with high-risk HR-positive *HER2*-negative eBC, PARPi should be considered as part of the treatment plan. | PARPi | 89 | 66∙4 | 87∙4 | A slight gap observed between real-world practice and recommendations of SCMs |
| d |  | Abstain | 14 | 10∙4 |  |  |
| e |  | Others | 3 | 0∙7 |  |  |
| **8.** |  | **For *BRCA*** **germline pathogenic variant carriers with metastatic HR+*/HER2*-negative, who have consumed hormonal options and previously received CT, my next treatment option would be** | | |  |  |
| a |  | CT | 7 | 5∙2 |  |  |
| b |  | CT + IO | 8 | 6∙0 |  |  |
| c | There is strong clinical evidence that PARPi delays disease progression in *BRCA* germline pathogenic variant carriers with metastatic *HER2*-negative BC who have previously been treated with CT in the neoadjuvant, adjuvant, or metastatic setting. | PARPi | 98 | 73∙1 | 87∙5 | Real-world practice concurs with the recommendations of SCMs |
| d |  | Abstain | 19 | 14∙2 |  |  |
| e |  | Others | 2 | 1∙5 |  |  |
| **9.** |  | **For *BRCA*** **germline pathogenic variant carriers with *HER2*+ eBC, besides *HER2*-directed therapy, I might consider PARPi.** | | |  |  |
| a | There is no current evidence to use adjuvant PARPi combined with anti-*HER2*–directed therapies in BRCA germline pathogenic variant carriers with *HER2*-positive eBC | Yes | 66 | 49∙3 | 92∙3 | A slight gap observed between real-world practice and recommendations of SCMs |
| b |  | No | 26 | 19∙4 |  |  |
| c |  | Not sure | 34 | 25∙4 |  |  |
| d |  | Abstain | 8 | 6∙0 |  |  |

BC: Breast cancer; CDK4/6i: Cyclin-dependent kinase 4/6 inhibitor; CRRM: contralateral risk reducing mastectomy; CT: Chemotherapy; eBC: Early breast cancer; ET: Endocrine therapy; eTNBC: Early triple-negative breast cancer; HCP: Healthcare practitioner; *HER2+*: Human epidermal growth factor receptor 2 positive; HR+: Hormone receptor positive; IO: Immunotherapy; PARPi: Poly ADP-ribose polymerase inhibitor; pCR: Pathological complete response; RRM: Risk-reducing mastectomy; SCM: Steering committee member; TNBC: Triple-negative breast cancer

Colour coding represents:

|  | Real-world practice concurs with the recommendations of SCMs |
| --- | --- |
|  | A significant gap observed between real-world practice and recommendations of SCMs |
|  | A slight gap observed between real-world practice and recommendations of SCMs |

**Supplementary Figure Legends**

**Supplementary Figure S1.** Country-wise distribution of healthcare practitioners

**Supplementary Figure S2.** Primary speciality of healthcare practitioners
