# Supplementary figures and images for: Part II: consensus statements and expert recommendations for BRCA-associated breast cancer in the Asia-Pacific region: clinical management
Source: Front Oncol. 2025 Jun 23;15:1507840. doi: 10.3389/fonc.2025.1507840 (PMC12230081; doi:10.3389/fonc.2025.1507840)

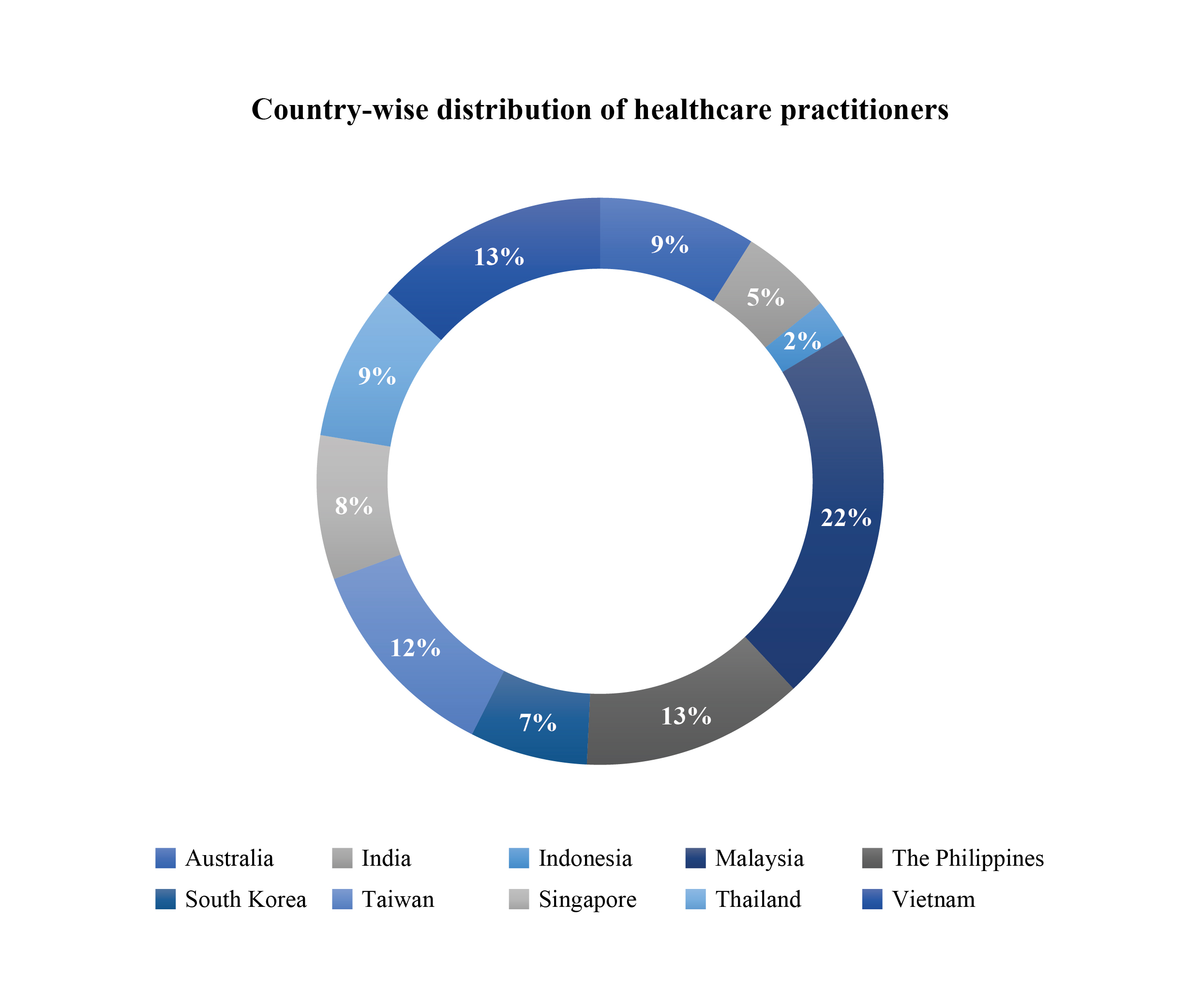

Supplement: Supplementary file 2 [file Image1.jpeg]

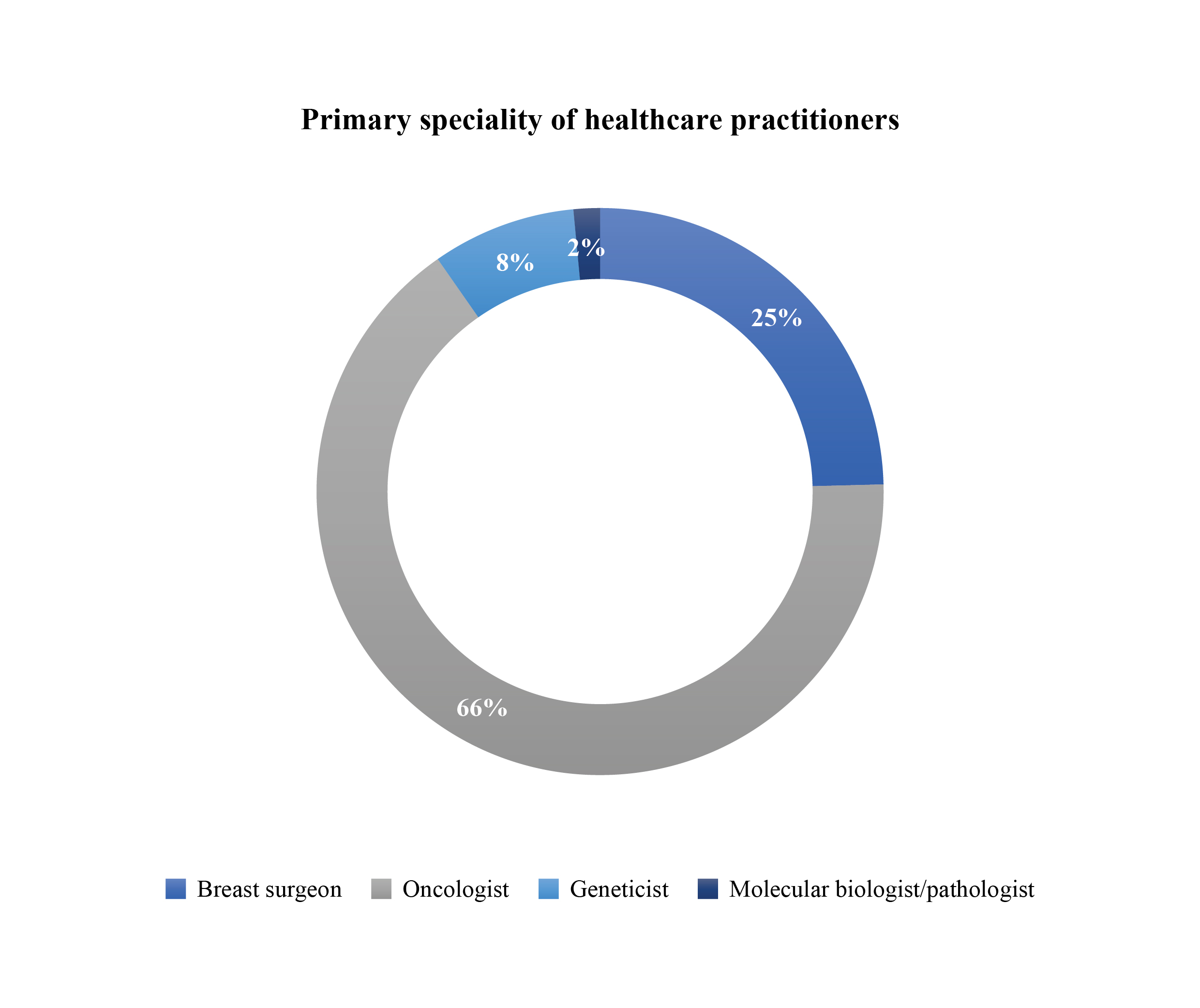

Supplement: Supplementary file 3 [file Image2.jpeg]
